# Screening and characterization of vaginal fluid donations for vaginal microbiota transplantation

**DOI:** 10.1038/s41598-022-22873-y

**Published:** 2022-10-26

**Authors:** Laura J. Yockey, Fatima Aysha Hussain, Agnes Bergerat, Alexandra Reissis, Daniel Worrall, Jiawu Xu, Isabella Gomez, Seth M. Bloom, Nomfuneko A. Mafunda, Julia Kelly, Douglas S. Kwon, Caroline M. Mitchell

**Affiliations:** 1 Department of Medicine, Massachusetts General Hospital, Boston, MA USA; 2 Ragon Institute of MIT, MGH and Harvard, Cambridge, MA USA; 3 Harvard Medical School, Boston, MA USA; 4 Department of Obstetrics and Gynecology, Massachusetts General Hospital, Boston, MA USA; 5 Division of Infectious Diseases, Massachusetts General Hospital, Boston, MA USA

**Keywords:** Bacterial infection, Reproductive disorders, Microbiology, Medical research

## Abstract

Bacterial vaginosis (BV), the overgrowth of diverse anaerobic bacteria in the vagina, is the most common cause of vaginal symptoms worldwide. BV frequently recurs after antibiotic therapy, and the best probiotic treatments only result in transient changes from BV-associated states to “optimal” communities dominated by a single species of *Lactobacillus*. Therefore, additional treatment strategies are needed to durably alter vaginal microbiota composition for patients with BV. Vaginal microbiota transplantation (VMT), the transfer of vaginal fluid from a healthy person with an optimal vaginal microbiota to a recipient with BV, has been proposed as one such alternative. However, VMT carries potential risks, necessitating strict safety precautions. Here, we present an FDA-approved donor screening protocol and detailed methodology for donation collection, storage, screening, and analysis of VMT material. We find that *Lactobacillus* viability is maintained for over six months in donated material stored at − 80 °C without glycerol or other cryoprotectants. We further show that species-specific quantitative PCR for *L. crispatus* and *L. iners* can be used as a rapid initial screening strategy to identify potential donors with optimal vaginal microbiomes. Together, this work lays the foundation for designing safe, reproducible trials of VMT as a treatment for BV.

## Introduction

Bacterial vaginosis (BV)—a syndrome characterized by vaginal discharge, odor, and discomfort—affects 30% of women worldwide^1^. It is associated with the presence of high diversity, *Lactobacillus*-deficient anaerobic vaginal microbiota, and it carries increased risk for a number of adverse sexual and reproductive outcomes, such as preterm birth, miscarriage, cervical dysplasia, and sexually transmitted infections, including HIV^2^. Antibiotic treatment, most commonly with metronidazole, reduces the absolute quantity of BV-associated microbes and temporarily improves symptoms, but BV recurrence is as high as 30–60% one month after completion of therapy^1,3,4^.

The two most prevalent and abundant vaginal *Lactobacillus* species are *Lactobacillus crispatus*, which is generally associated with beneficial health outcomes, and *Lactobacillus iners*, which is linked to microbial community instability, adverse outcomes, and transition to diverse, BV-like communities^5–8^. Delivery of exogenous *L. crispatus* to the vagina as a live biotherapeutic has been studied for prevention of recurrent BV, but multiple studies have shown only modest benefit^9–11^. In a Phase 2b trial of a probiotic consisting of a single strain of *L. crispatus* (LACTIN-V), after 5 daily doses and twice-weekly dosing for 11 weeks, 30% of women in the treatment arm had BV recurrence immediately after cessation of treatment, with an increase to 39% after an additional 12 weeks without treatment^12^. Single strain probiotics likely demonstrate only partial efficacy for a variety of reasons: one hypothesis being that the diverse array of bacteria, phages, and small molecules comprising the entirety of a healthy vaginal ecosystem, along with the interactions between them, may be required to support lactobacilli and sustainably shift the community. Similar hypotheses have been proposed to explain the success of fecal microbiota transplants (FMT) for the treatment of recurrent *Clostridium difficile* colitis in the gut^13–16^.

Vaginal microbiota transplantation (VMT), the transfer of whole vaginal fluid from a healthy person with high *Lactobacillus* abundance, has been proposed as a solution to establish a healthy vaginal microbiome after antibiotic treatment for BV. In 2019, the first report of VMT in humans demonstrated remission of BV in 4 of 5 women treated multiple times over 2 years with antibiotics followed by a small amount of fresh vaginal fluid^17^. The authors reported no significant side effects and no serious adverse events. While the lack of adverse outcomes reported in this study is reassuring, the small study size and lack of a placebo arm make it difficult to interpret whether VMT provided an added benefit over antibiotics alone. A separate group published a more extensive proposal for characterization of donors to ensure safety in VMT and described properties of collected vaginal fluid^18^. However, for VMT to be a viable treatment for BV, strategies for efficient identification of suitable donors, as well as optimized methods for collection, screening, and storage of donated material are needed.

Here we describe the recruitment and enrollment of three vaginal fluid donors under the first Food and Drug Administration (FDA)-approved Investigational New Drug (IND) protocol for VMT. In addition to screening for general donor health and the absence of infectious diseases in the donors, we outline additional measures for safety screening of donors and donated material. Furthermore, we demonstrate comparability of donations and aliquots reserved for analysis and show stability of viable *Lactobacillus* in donations over time. This protocol, and the described donations meeting all quality metrics, will be used in a planned randomized trial of VMT to promote a *Lactobacillus*-dominant vaginal microbial community in patients diagnosed with recurrent BV.

## Results

### Donation collection optimization

**Figure 1:**
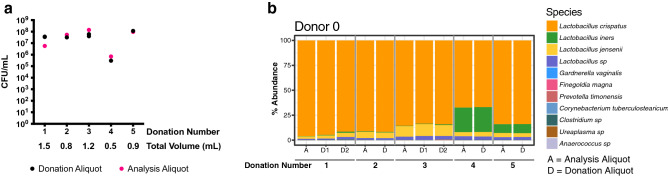
Microbial community profiling and absolute quantification of pilot donation fluid. (**a**) *Lactobacillus* colony forming unit (CFU) quantification for each donation and analysis aliquot. Total donation volume is listed below donation numbers. (**b**) Microbial species in donation (“D”) and analysis (“A”) aliquots of pilot donor (Donor 0) samples using bacterial 16S rRNA amplicon sequencing. D1 and D2 refer to two different donation aliquots from the same donation collection.

We enrolled a pilot donor (Donor 0) to initially optimize sample collection and processing procedures, and to confirm the feasibility of the protocol. Vaginal fluid was collected using a disposable menstrual cup and donation material from Donor 0 was homogenized with sterile saline (see Methods). The donation material was then split into several “analysis aliquots,” which were stored at − 80 °C to test for *Lactobacillus* viability over time, and a remaining “donation aliquot” to be used for eventual transplantation. Donor 0 provided five donations over nine days, with a median of 400 μL of vaginal fluid per donation (range < 100–1000 μL). After 13 months stored at − 80 °C, *Lactobacillus* Colony Forming Unit (CFU) counts were similar in the two types of aliquots: median 3.7 × 10^7^ CFU/mL in the donation aliquot compared to 5.3 × 10^7^ CFU/mL in an analysis aliquot (Fig. 1a).

Microbial community composition of the donation and analysis aliquots by bacterial 16S rRNA amplicon sequencing revealed that the bacterial community profile of both aliquot types was nearly identical at each timepoint (Fig. 1b). The relative abundances of *L. crispatus* and *L. iners* varied over time across the five donations, but *L. crispatus* was consistently at a higher relative abundance (66.9–96.0%) than *L. iners* (0.35–24.9%). Non-*Lactobacillus* taxa represented < 0.8% of total relative abundance for each sample. Based on the lower CFU count in the single donation with the lowest volume, we set a conservative minimum threshold of 700 μL for a donation to be acceptable for use. However, the reduction in CFU counts for this sample was likely in part due to the higher relative abundance of *L. iners*, which typically does not grow on the MRS (deMan, Rogosa, and Sharpe) agar used for *L. crispatus* culturing.

### Donor enrollment

**Figure 2:**
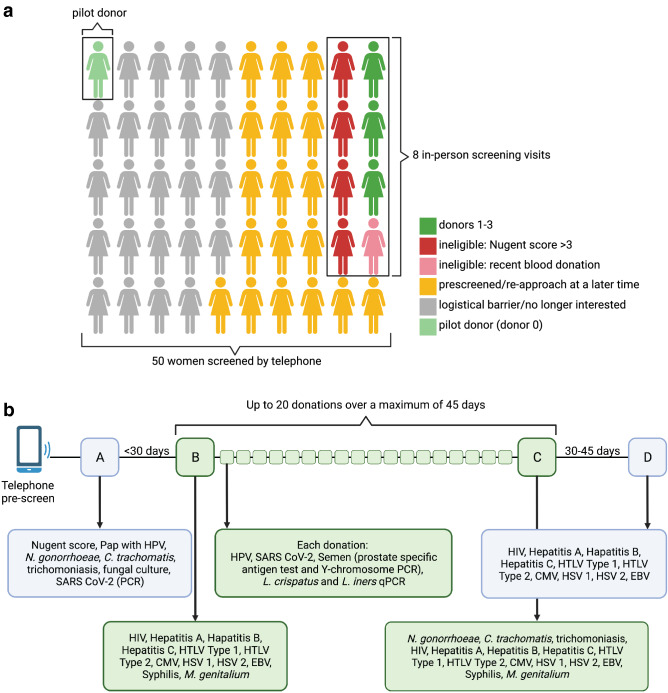
Donor screening and donation schedule. (**a**) 50 total women, including the pilot donor (light green), were pre-screened over the phone. Eight potential donors completed an in-person screen and testing, three of whom went on to become donors (dark green), four of whom were ineligible due to high Nugent scores (red), and one of whom was ineligible due to a recent blood donation and no subsequent follow-up (pink). Twenty-three potential donors (grey) were uninterested in following up or were disqualified for logistical reasons (e.g., travel), 18 completed pre-screening and asked for later follow up (yellow). (**b**) Donation screening and schedule with detailed testing for donors and donations before, during, and after donating.

After demonstrating the feasibility of donation collection, we received FDA approval for our Investigational New Drug protocol (#018173). Between March 2019 and November 2020, we screened 49 additional women by telephone and conducted 8 in-person screening visits to enroll three donors (Donors 1–3) (Fig. 2a). Women were considered for the study if they were premenopausal, had a Nugent score of 0–3, agreed to abstain from sexual activity during the entire donation period, and denied any history of BV. An extensive set of inclusion and exclusion criteria were used for final screening (see Methods). Four potential donors failed screening because of a Nugent score > 3, and one person could not have screening labs drawn due to a recent blood donation and did not return for a follow-up visit to complete screening.

### Donor screening

All three donors identified as white, cis-gender women and reported a preference for male sexual partners. Donors 1 and 3 were using oral contraceptives and Donor 2 was using a Mirena IUD for contraception. A full list of screening tests is outlined in Table 1, which include tests to screen for underlying medical comorbidities (acceptable ranges in Table 1 and Table S1). Donor demographics including age, race, and BMI are included in Table S2. Enrolled donors went through extensive testing for potentially transmissible infections at enrollment, at the final donation visit, and 30–45 days after the final donation (Fig. 2B). This ensured identification of an incident infection during the entire donation period. Participants agreed to remain sexually abstinent during the donation period. Each individual donation was tested for prostate specific antigen (PSA) at the time of processing to confirm the absence of semen, and analysis aliquots for each donation were tested for HPV and again for sperm using a Y-chromosome-specific PCR assay. Starting in June 2020, all donors underwent nasal swab PCR testing for SARS-CoV-2 prior to enrollment, were screened for symptoms before each donation, and each donation was tested for SARS-CoV-2 by RT-PCR.

**Table 1:** Overview of donor screening tests.

Test	Acceptable range	Timing
Vaginal pH	< 4.8	Screening
Wet mount	No yeast, trichomonas	Screening
Vaginal Gram stain	Nugent < 3	Screening
Urine pregnancy test	Negative	Screening
Complete blood count	WBC: 4.5–11 K/μL Hematocrit: 30–46% Hemoglobin: 12–16 g/dL Platelets: 100–400 K/μL	Screening
Basic metabolic panel	Values < 1.2 × upper limit of normal*	Screening
Liver function tests	Values < 1.2 × upper limit of normal*	Screening
Hemoglobin A1C	4.3–6.4%	Screening
Pap smear	No abnormalities	Screening
Human papillomavirus DNA	Negative for high-risk types	Screening Each donation
Urine culture	Negative	Screening
Urine toxicology screen	Negative	Screening
Semen (PSA card)	Negative	Each donation
Nucleic acid amplification tests for *Neisseria gonorrhoeae, Chlamydia trachomatis, Trichomonas vaginalis, Mycoplasma genitalium*	Negative	Screening Final donation
Treponemal test for syphilis (Trep-Sure)	Negative	
HIV 1 and 2 antibody/antigen, HIV viral load	Negative	Screening Final donation 30–45 days after final donation
Hepatitis A IgM	Negative	
Hepatitis B surface antigen	Negative	
Hepatitis B core antibody, Total and IgM	Negative	
Hepatitis C antibody	Negative	
Human T-lymphotrophic Virus, type 1 and 2	Negative	
Cytomegalovirus IgG and IgM	IgM negative If CMV IgG positive, CMV PCR performed on each donated vaginal fluid aliquot	
Herpes Simplex Virus type 1 and 2 IgG antibodies	HSV2 IgG negative If HSV1 IgG positive, HSV PCR performed on each donated vaginal fluid aliquot	
Epstein-Barr virus heterophile antibody (Monospot)	Negative	

*Normal ranges in Table S1.

### Donation collection for potential VMT

Donor 1 provided eight donations over 12 days, with a median volume of 0.4 mL (range 0.1–0.8 mL) and a median weight of 1.42 g (range 1.0–1.8 g) (Fig. S1). Donor 2 provided 20 donations over 37 days, with a median volume of 0.75 mL (range 0.3–1.1 mL) and a median weight of 1.3 g (range 1.1–2.0 g). Donor 3 provided 14 donations over 40 days, with a median volume of 0.55 mL (range 0.4–0.9 mL) and a median weight of 1.1 g (range 0.5–1.5 g). All donations had a pH < 4.8 and all donors had a Nugent score < 4 at the time of donation.

### Donation characteristics for potential VMT

**Figure 3:**
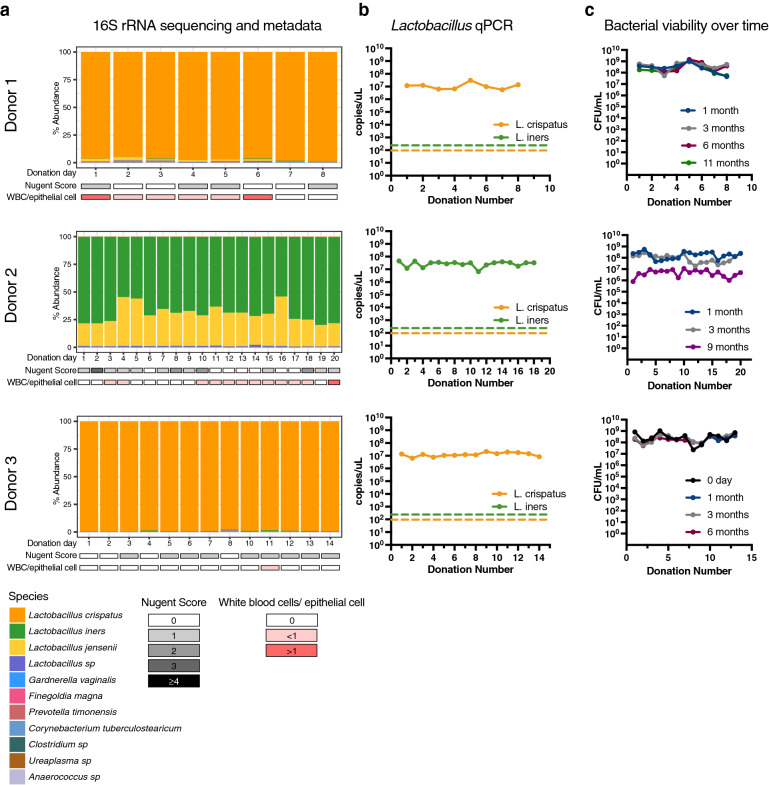
Microbial community profiles of VMT donation samples. (**a**) Microbial community and metadata. Relative abundance of the bacterial community in the donation material was determined using 16S rRNA amplicon sequencing for each donation. Nugent score and white blood cell (WBC)/epithelial cell ratio are presented below each donation. (**b**) Absolute quantification of *L. crispatus* and *L. iners* in donation samples using species-specific qPCR. Detection limit of the assay is depicted by dashed lines. (**c**) Donation stability measured as *Lactobacillus* CFUs streaked and counted on MRS agar at long-term intervals post freezing.

For Donor 1, the Nugent scores for all donations ranged from 0–1 (Fig. 3a). For most gram stain slides, there were no white blood cells (WBCs) present or < 1 WBC/epithelial cell, but > 1 WBC/epithelial cell was seen on 2 donation samples (1 and 6) (Fig. 3a). High numbers of white blood cells often indicate a vaginal infection, but this donor had negative testing for known infections. For Donor 2, the Nugent score ranged from 0 to 3. Similar to Donor 1, there were no WBCs present or < 1 WBC/epithelial cell except for 1 day (sample 20). Donor 3 had Nugent scores of 0–1, and no samples had > 1 WBC/epithelial cell.

To determine the bacterial community composition of the VMT donors, we used 16S rRNA amplicon sequencing along with species-specific qPCR assays for *L. crispatus* and *L. iners*. We found Donor 1 and Donor 3 were *L. crispatus* dominant for the entire donation period (Fig. 3a). Donor 2, however, had varying levels of *L. jensenii* and *L. iners*, which together made up nearly the entire community for all donation samples.

Using species-specific qPCR assays, *L. crispatus* and/or *L. iners* were found to be almost mutually exclusive in these donors. In Donor 2, qPCR results showing high quantities of *L. iners* and no detectable *L. crispatus* demonstrated that despite normal Nugent scores, the primary *Lactobacillus* species was not *L. crispatus*, but rather *L. jensenii*. Additionally, despite *L. jensenii* having a higher relative abundance in the 16S rRNA sequencing, the absolute quantity of *L. iners* in the donations from Donor 2 was high (Fig. 3b).

### *Lactobacillus* viability after untreated storage at − 80 °C

Although a previous study used fresh donations for VMT, for long term use and scalability of the donated material, optimal storage protocols are needed. We aimed to maximize viability of the bacteria in the samples over time and to minimize any modifications to the material. We thus froze analysis aliquots of each donation without the addition of glycerol or other cryoprotectants at − 80 °C and used the aliquots to quantify the stability of *Lactobacillu*s. To measure viable *Lactobacillu*s CFUs after storage at − 80 °C, aliquots from each donation were serially diluted and plated on *Lactobacillus-*selective MRS agar plates (Fig. 3c). For Donor 1, *Lactobacillus* viability (CFU/mL of sample) was stable between 30 days (median 2.8 × 10^8^, range 4.5 × 10^7^–1.0 × 10^9^), 3 months (median 5.2 × 10^8^, range 5.3 × 10^7^–9 × 10^8^), 6 months (median 3.7 × 10^8^ range 1.2 × 10^8^–1.5 × 10^9^), and 11 months (median 1.7 × 10^8^ range 5.5 × 10^7^–1.1 × 10^9^) after collection. For Donor 2, *Lactobacillus* viability (CFU/mL) was initially stable between 30 days (median 2.0 × 10^8^, range 4.7 × 10^7^–5.6 × 10^8^) and 3 months (median 1.1 × 10^8^, range 1.9 × 10^7^–2.9 × 10^8^) but declined at 9 months (median 5.2 × 10^6^, range 8 × 10^5^–1.1 × 10^7^). This decline may be attributed to the difference in makeup of the community and associated differences between *L. crispatus* compared to the mixture of *L. jensenii* and *L. iners*, the latter of which does not typically grow on MRS. Donor 3 was similar to Donor 1, with stable CFU counts when comparing data at collection (median 1.8 × 10^8^ CFU/mL, range 1.5 × 10^7^–5.8 × 10^8^ CFU/mL), 3 months (median 1.6 × 10^8^ CFU/mL, range 3.6 × 10^7^–2.8 × 10^8^ CFU/mL) and 6 months (median 1.2 × 10^8^ CFU/mL, range 3.4 × 10^7^ to 3.8 × 10^8^ CFU/mL). This suggests that donations with *L. crispatus* dominant communities can be stored without need for cryoprotectants.

## Discussion

While VMT has been proposed as a potential strategy to prevent recurrent BV, detailed safety, feasibility, and efficacy testing is needed before this can be considered as an option for clinical care. Here we describe a protocol for extensive safety screening and collection of vaginal fluid donations for VMT. In our screening strategy, we test participants before, during, and after the donation period to ensure that they do not have or did not recently become infected with common sexually transmitted pathogens. We also confirm absence of semen or sperm in donation materials. We show that this collection protocol is feasible, with our enrollment of 3 donors, who together provided a total of 42 doses of at least 700 μL each. Additionally, our processing protocol resulted in donation aliquots with viable lactobacilli over several months of storage at – 80 °C, and our analysis aliquots reflected the characteristics of the donation aliquots, which will be important in characterizing successful donations.

While all three donors showed stable *Lactobacillus* viability at − 80 °C storage up to 3 months, there was a 1–2 order of magnitude decline in viability of the samples from Donor 2 at 9 months. For this donor, the 6-month time point was not performed due to the COVID-19 pandemic. Molecular analysis demonstrated that this donor had a majority of *L. jensenii* and high absolute quantities of *L. iners*, in contrast to the other two donors who had high relative and absolute abundance of *L. crispatus*. It is possible that the different *Lactobacillus* species survive freezing differently, or that another property of the vaginal fluid that differed between the donors (e.g., glycogen content) contributed to this discrepancy in long-term viability. For our first randomized trial of vaginal fluid transplant, our goals are to 1) prioritize recipient safety, and 2) to limit our manipulation of the vaginal fluid so that we are best able to identify features associated with benefit (or lack thereof). For this reason, we did not include cryoprotective solutions when processing the donations—we were concerned that these might increase the risk for vulvovaginal candidiasis in recipients and that they might obscure our ability to characterize donation characteristics. Based on our results, we feel that use of cryoprotectants is not necessary if a minimum concentration of 1 × 10^6^ CFU/mL of *L. crispatus* is present. Additionally, because of the differences in viability we decided only to include donations that are *L. crispatus* dominant.

Our initial intent was to do as little characterization of the microbiota as possible prior to selection of donation material, to remain agnostic to what might be the beneficial component or optimal composition of vaginal fluid. However, the difference in viability of the donation material highlights a challenge in using Nugent score to screen donors. Although the Nugent method is efficient and quick, it led to the enrollment of a donor with a *L. jensenii* dominant community with a high absolute quantity of *L. iners*. This donation material was less stable when stored at − 80 °C than *L. crispatus-*dominant donations from the two other donors. Using species-specific qPCR assays for *L. crispatus* and *L. iners* to test the donation material clearly distinguished the two optimal donors from the donor with a higher proportion and quantity of *L. iners*. Based on these results, we propose screening future donations with qPCR and excluding donors or donations with competing absolute abundances of *L. iners* compared to *L. crispatus*. We have chosen to focus on these two species as they are the most commonly identified dominant lactobacilli in the vagina, and because of the differences in viability seen when levels of *L. iners* are high and *L. crispatus* is absent. We suggest the concentrations of *L. crispatus* to be at least three orders of magnitude higher than that of *L. iners.* The proposed qPCR strategy is faster and cheaper than completing 16S rRNA amplicon sequencing for each donation and will allow rapid assessment of the suitability of donations while awaiting the more comprehensive characterization of the microbiota that will occur in the analysis phase of the study.

Our safety screening of donors and donations was extensive. Of note, no individual donation tested positive for HPV, suggesting no intermittent shedding of HPV in the donors, all of whom had a negative HPV test with the screening Pap smear. Additionally, no individual donations tested positive for PSA or Y-chromosome, suggesting donors also abstained from penile-vaginal intercourse during the donation period. The assay can detect semen to a 1:512 dilution and has been shown to detect semen for up to 5 days after a sexual encounter^19^. This extensive testing for sperm/semen minimizes risk of inducing pregnancy in the recipient and minimizes the possibility of acquisition of sexually transmitted infections. Furthermore, viable sperm are unlikely to survive the freeze/thaw process, adding an additional level of safety.

VMT is a promising strategy to promote a *Lactobacillus*-dominant vaginal microbiota in people with recurrent BV. Studies of VMT will also provide a novel opportunity to identify host and microbial determinants of *Lactobacillus* colonization and persistence. This report describes a feasible, reproducible protocol for the collection of donated vaginal fluid with safeguards to ensure recipient safety*.* Our protocol differs from the published report of VMT in our method of collection, storage of frozen donations until multiple safety tests have been completed, and creation of analysis aliquots directly from the donated material (rather than swabs collected at the same time). The minimal processing in our protocol maintains viability of lactobacilli and will allow us to assess the impact of whole vaginal fluid on recipient microbiota and mucosal immunity, without being confounded by the addition of cryoprotectant or stabilizing compounds. With the proposed minimal processing of donated material, we consistently count between 10^8^ and 10^9^ *Lactobacillus* CFU/mL, which means a dose of 700 μL will deliver between 10^7^ and 10^9^ CFU. This concentration is comparable to the dose of Lactin-V, the *L. crispatus* live biotherapeutic used in a Phase2b clinical trial in 2019^12^. Finally, the retention of analysis aliquots will facilitate identification of features linked to successful outcomes (e.g., specific strains associated with success).

Despite the high prevalence of BV, and lack of durable treatment response with current therapies, there has not been a fundamentally new therapy for BV in decades. VMT is a strategy to gain insights into determinants of vaginal colonization with beneficial lactobacilli and contribute to the development of novel strategies for BV treatment and prevention. Successful collection, testing, and storage of donated vaginal fluid is a first step toward that goal.

## Methods

### Donor recruitment

Potential donors were recruited by website postings, flyers, recruitment from previous studies, and advertisements. Posters were placed in sexual health and gynecology clinics as well as around the campuses of local universities.

We defined eligible donors as healthy, pre-menopausal (age 18–40), non-pregnant individuals who had never had a diagnosis of BV, were sexually experienced (thus have had the opportunity to acquire BV), and willing to abstain from any sexual activity for the duration of the screening and donation period. At initial screening, a donor needed to have a vaginal fluid pH < 4.8 and a Nugent score between 0 and 3. Potential donors underwent screening based on the American Association of Blood Banks (AABB) donor questionnaire for exposure to infectious agents. As part of the AAB questionnaire, potential donors are also asked about their medications and excluded if taking medications not deemed safe for blood donors. After June 2020, the protocol was amended to exclude participants that declared having symptoms of COVID-19 during the phone screen. Exclusion criteria also include: history of non-BV cervicovaginal infections in the past year; abnormal Pap smear or positive test for high risk HPV within the past year; use of probiotics within prior 30 days; oral or vaginal antibiotics within prior 3 months; history of genital herpes; abnormal findings on physical exam; personal travel in prior 6 months to a country or territory with active Zika outbreak (based on CDC guidelines) or unprotected sex in the preceding month with a partner who traveled to an area with active Zika in the prior 6 months.

### Donor screening

Donors were tested for viral, bacterial, and fungal infections (HIV, Hepatitis A, B, C, Herpes Simplex Virus (HSV), Cytomegalovirus (CMV), Syphilis, Human T-lymphotrophic Virus (HTLV 1 and 2), Epstein-Barr virus (EBV), Gonorrhea, Chlamydia, Trichomonas, Candida, and *Mycoplasma genitalium)* prior to, at the end of, and 30 days after the donation period (Table 1, Fig. 2b). In addition, at enrollment, donors had a complete blood count, basic metabolic panel, hemoglobin A1C, and liver function tests checked to screen for underlying medical comorbidities. After June 2020, all donors also underwent nasal swab PCR testing for SARS CoV-2 prior to their enrollment appointment. Urine toxicology screen for illicit drugs, pregnancy test, and Pap smear with HPV testing was also performed at the time of enrollment. All testing was performed in the CLIA-certified hospital lab, according to routine clinical testing procedures. Any positive test (with the exception of HSV1 IgG or CMV IgG) resulted in exclusion. Donor 1 had positive tests for HSV1 IgG and CMV IgG. Our protocol specifies that in those cases each donation will be tested for HSV or CMV with PCR and excluded if positive. However, due to the COVID-19 pandemic these donations expired before confirmatory testing was performed. Donors consented to abstain from sexual contact (including oral, vaginal, anal giving or receiving) during the period of donation.

### Donation collection and testing

The donation period started 3–10 days after the last day of a participant’s menstrual period. Per protocol, donors could provide up to 20 donations in a 45-day window. To donate vaginal fluid, participants inserted a disposable menstrual cup (Softdisc) 6–12 h prior to arriving at the clinical site, and removed the cup on site, where study staff processed the donation immediately.

Vaginal secretions were collected from the Softdisc by centrifugation in a sterile 50 mL conical tube at 810×*g* at 4 °C for 10 min, diluted with 500 μL of sterile saline and sheared with a blunt 16-gauge needle to homogenize the solution. A swab of material left on the Softdisc was used to make a slide for Nugent scoring and to test for semen using a prostate-specific antigen (PSA) card^19^. Each donation was aliquoted into treatment doses (at least 700 μL), and several 50 μL analysis aliquots to perform longitudinal *Lactobacillus* viability testing and to characterize the components of a successful transplant dose.

Analysis aliquots were used to test for the presence of semen using a Y-chromosome PCR^20^, and for high risk HPV using BD Oncoclarity test in the hospital clinical lab^21^. *Lactobacillus* CFU counts were quantified by thawing a separate analysis aliquot, performing serial dilutions, and plating 10 µl of diluted samples (1:10^3^–1:10^6^) in triplicate on MRS agar plates (Hardy Diagnostics) under anaerobic conditions (5% H2/5%CO2/90%N2 anaerobic mixed gas). Plates were incubated at 37 °C in a Bactron anaerobic chamber for two days prior to counting. The number of colonies was counted manually for each dilution with less than 100 colonies per 10 µl replicate.

DNA was extracted from an additional analysis aliquot using a manual phenol chloroform extraction and used for sequencing of the V4 region of the 16S rRNA gene as previously described^22^. Single-end sequencing was performed on an Illumina MiSeq using a v2 300-cycle kit. Data was demultiplexed using a custom python script and denoised using DADA2^23^. Taxonomy of amplicon sequence variants was assigned using RDP^24^ in addition to manually curated assignments. Finally, data was analyzed in R using phyloseq^25,26^.

Quantification of *L. crispatus* and *L. iners* was performed using TaqMan qPCR assays as previously described^27,28^. Briefly, 5uL of DNA was used in a total 20uL reaction with species-specific primers and probe over 45 amplification cycles. A plasmid standard curve was used to calculate quantity of 16S rRNA gene copies per reaction and data were normalized to the volume of donation sample. Specific conditions are provided in Table S3.

### Ethical parameters

All participants signed informed consent, and all procedures were performed per the human subjects approved protocol (Partners Human Subjects Committee, 2018P000057).

## Supplementary Information

Supplementary Information.

## Data Availability

Sequencing data is available from the NCBI Sequence Read Archive (BioProject ID: PRJNA812510). Code for data processing of 16S amplicon data available from Zenodo (https://doi.org/10.5281/zenodo.6374205).
