# Supplementary material for: Screening and characterization of vaginal fluid donations for vaginal microbiota transplantation
Source: Sci Rep. 2022 Oct 26;12:17948. doi: 10.1038/s41598-022-22873-y (PMC9606370; doi:10.1038/s41598-022-22873-y)
Supplement: Supplementary file 1 — Supplementary Information. [file 41598_2022_22873_MOESM1_ESM.pdf]

Supplementary Information

Screening and Characterization of Vaginal Fluid Donations for Vaginal Microbiota Transplantation

Laura J. Yockey & Fatima Aysha Hussain, et al.
Corresponding author: Caroline M. Mitchell (caroline.mitchell@mgh.harvard.edu)

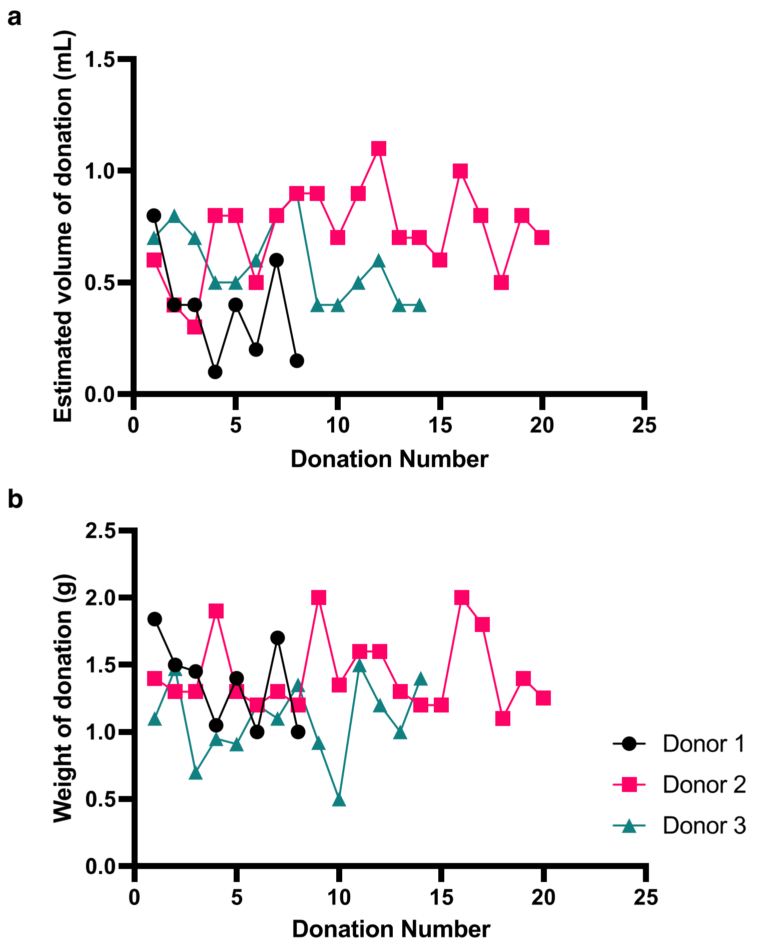

Figure S1: Volume **(a)** and weight **(b)** of donated vaginal fluid varied donation to donation and donor to donor.

Table S1: Screening lab acceptable values

Measurement	Reference range
White Blood Count	4.5-11K/μL
Hematocrit	36-46%
Hemoglobin	12-16 g/dL
Platelets	150-400K/μL
Hemoglobin A1C	4.3-6.4%
Sodium	135-145 mmol/L
Potassium	3.4-5.0 mmol/L
Chloride	98-108 mmol/L
CO2	23-32 mmol/L
BUN	8-25 mg/dL
Creatinine	0.6-1.5 mg/dL
AST	9-32 U/L
ALT	7-33 U/L
Alkaline phosphatase	30-100 U/L
Total bilirubin	0-1 mg/dL
Direct bilirubin	0-0.4 mg/dL

Table S2: Donor Demographics

Donor Number	Age (years)	Race/ethnicity	BMI
Pilot	36	White/Not Hispanic or Latino	34.5
1	28	White/Not Hispanic or Latino	23.3
2	25	White/Not Hispanic or Latino	23.1
3	32	White/Not Hispanic or Latino	20.7

Table S3: Quantitative PCR conditions

Assay	Primer and probe sequences	PCR conditions	Per 20uL reaction
L. crispatus	989F TCTTGACATCTAGTGCCATTTGT<br>1055R TGCACCACCTGTCTTAGC<br>FAM-TAMRA<br>CCGAAGGGAACTTTGTAGCGCGAC	50C x 2 min<br>95C x 10 min<br><br>45 cycles:<br>95C x 15s<br>65C x 39s<br>72C x 20s	Primers 0.8 uM<br>Probe 0.15uM<br>Taq Fast Advanced Master Mix 10uL<br>Sample 5uL
L. iners	165F GATGCTAATACCGGATAAYAACAGAT<br>241R CACCGCAGGTCCATCCAAGA<br>FAM-TAMRA<br>TGCCTATCAACTGTTTAAAAGATGGTTCT	50C x 2 min<br>95C x 10 min<br><br>45 cycles:<br>95C x 19s<br>55C x 39s<br>72C x 20s	Primers 0.8 uM<br>Probe 0.15uM<br>Taq Fast Advanced Master Mix 10uL<br>Sample 5uL
As described in Srinivasan et al. and McClelland et al.[27,28]			
